# Combined low-dose isotretinoin and long-pulsed nd: YAG laser in the treatment of post-acne erythema

**DOI:** 10.1007/s00403-024-03143-5

**Published:** 2024-06-08

**Authors:** Waleed Albalat, Rana Ehab, Mohamed Hussein AbouHadeed, Tarek N. Abd Allah, Reham Essam

**Affiliations:** 1 Department of Dermatology, Venereology and Andrology, Faculty of Medicine, Zagazig University, Zagazig, Egypt; 2 Research Department of Dermatology and Venereology, Medical Research and Clinical Studies Institute, National Research Centre, Giza, Egypt; 3 Department of Dermatology and Venereology, Faculty of Medicine, Misr University, Giza, Egypt

## Abstract

Post-acne erythema (PAE) is a bothering skin condition that emerges from inflammatory acne and persists after its resolution. It is characterized by telangiectasia and erythematous macules. the role of 1064-nm Nd: YAG when combined with low-dose isotretinoin in the acne erythema treatment. forty-eight PAE patients were involved in the study. They were divided into two groups; group (A) patients administering a low dose of oral isotretinoin (10 mg/day) and underwent a total of six two-week interval sessions of 1064 ND-YAG laser treatment, group (B) patients administering a low dose of oral isotretinoin (10 mg/day) only. All adverse effects experienced during the course of therapy were documented, and photos were taken before the start of the treatment and following the end of the treatment duration. Following the completion of the therapeutic intervention, a significant improvement in clinical condition was observed in both groups, with more improvement in group (A) compared to group (B) as evidenced by a notable improvement in the score on the Clinician Erythema Assessment Scale (CEAS) and also a significant decrease in the mean value of optical density of the erythema. combined 1064-nm Nd: YAG with low-dose isotretinoin may be an efficient and secure line in the PAE treatment. Also, the combined therapy had superior results when compared to low-dose isotretinoin alone.

## Introduction

Acne vulgaris is a prevalent dermatological issue that impacts approximately 9.4% of the worldwide population [1]. Acne usually occurs among individuals during the period of adolescence and early adulthood [2]. It is developed as a result of several key factors, including an inflammatory condition of the pilosebaceous follicle, follicular hyperkeratinization, elevated sebum production, and colonization by *Propionibacterium acne*. These elements collectively contribute to the pathogenesis of cutaneous acne lesions [3].

Post-acne erythema (PAE) emerges from inflammatory acne and persists after its resolution. It is characterized by telangiectasia and erythematous macules [4]. It is related to the secretion of inflammatory cytokines like interleukin (IL)-6 and tumor necrosis factor (TNF)-α. Intracapillary aggregations of erythrocytes and microcapillary dilatation caused by the healing process in the papillary dermis play crucial roles in the development of PAE. Additionally, as a wound heals, the epidermis thins, making dilated microcapillaries more visible [5, 6].

Investigations on the therapeutic effectiveness of various laser modalities for treating acne erythema have been performed. One of the effective therapeutic options has been shown to be the pulsed dye laser (PDL), 585–595 nm [7, 8], Q-Switched Neodymium: yttrium-aluminum-garnet (Nd: YAG) laser [9], erbium glass fractional laser [10], and intense pulsed light (IPL) devices [11].

Using a long-pulsed Nd: YAG laser with 1064 nm has become prevalent in skin rejuvenation. However, numerous studies and case reports have provided compelling evidence validating the effectiveness of this laser in acne treatment [12]. The therapeutic effects on acne lesions are thought to be attributed to several mechanisms, including selective photothermolysis of vessels, TGF-β upregulation, interleukin-8 (IL-8) reduction, and Toll-like receptors-2 (TLR-2), and thermal eradication of sebaceous glands [13–15].

Isotretinoin is the cornerstone of acne treatment [16]. Certainly, the negative consequences associated with this efficient medication discourage physicians from promptly recommending its use, especially with high doses [17]. Based on recent research findings, it has been suggested that the administration of lower doses, specifically at a rate of 0.25 mg/kg/day, may result in a reduction in the occurrence of adverse effects [18] as well as a decrease in flares [19]. Our therapeutic choices to achieve better control have increased with the authorization of the application of oral isotretinoin for use in conjunction with other interventions [20]. 

This study was done to suggest the possible role of long-pulsed Nd: YAG laser with 1064 nm when combined with low-dose Isotretinoin in the treatment of the bothering post-acne erythema.

## Patients and methods

The present study was carried out on 48 individuals who had been clinically diagnosed with post-acne erythema and had exhibited resistance to traditional treatments of acne over a period of six months prior to the study. The protocol underwent submission and subsequent approval by the Institutional Review Board (IRB). Each participant was assigned an informed written consent. The study excluded individuals who were concurrently using acne medications, had a previous history of photosensitivity reactions, had compromised hepatic function, had preexisting hyperlipidemia, were pregnant, intending to get pregnant during the treatment period, or had a medical history of hypertrophic scar or keloid.

The study involved administering a low dose of oral isotretinoin (10 mg/day) to a specific group of patients (group B). Other patients underwent six sessions of 1064 Nd: YAG laser treatment (Deka motous AY) with specific parameters: 150 J/cm2 energy density, 20–25 milliseconds pulse duration, and a 5 mm spot size, in combination with a low dose of oral isotretinoin (10 mg/day). The laser sessions were conducted at intervals of two weeks.

Any side effects during therapy were recorded, and photos were taken prior to the treatment, before each session, and following the treatment period.

The images were captured using a digital camera and subsequently assessed by a panel of three dermatologists. A scoring system consisting of four categories was employed to assess the degree of improvement in erythema. These categories were defined as follows: mild improvement, ranging from 1–25%; moderate improvement, ranging from 26–50%; good improvement, ranging from 51–75%; and excellent improvement, ranging from 76–100%. The Clinician Erythema Assessment Scale (CEAS) Table 1[21] was utilized to evaluate the erythema levels prior to and following treatment. Additionally, the optical density was measured using ‘image J’ 1.49 V/Java 1.6.0-244 (National Institute of Health, USA).

**Table 1:** Clinical erythema assessment scale

Score	Clinician erythema assessment scale	
0	Clear	Skin with absence of the erythema signs
1	Almost clear	A minor degree of erythema
2	Mild	Distinct redness
3	Moderate	Marked redness
4	Severe	Fiery redness

In instances where discrepancies occurred in the evaluations conducted by the dermatologists, a mean score was computed and subsequently employed for the purposes of analysis.

## Results

The present study is a randomized controlled trial that enrolled 48 participants who were recruited for the research inquiry, they were randomly distributed into two equal groups. twenty-eight patients (58.3%) had Fitzpatrick skin type III and 20 patients had Fitzpatrick skin type IV.

At the end of the full treatment period, the mean CEAS and the mean value of the optical density of the erythema significantly decreased when compared to baseline in both groups (A) and (B).

**Fig. 1:**
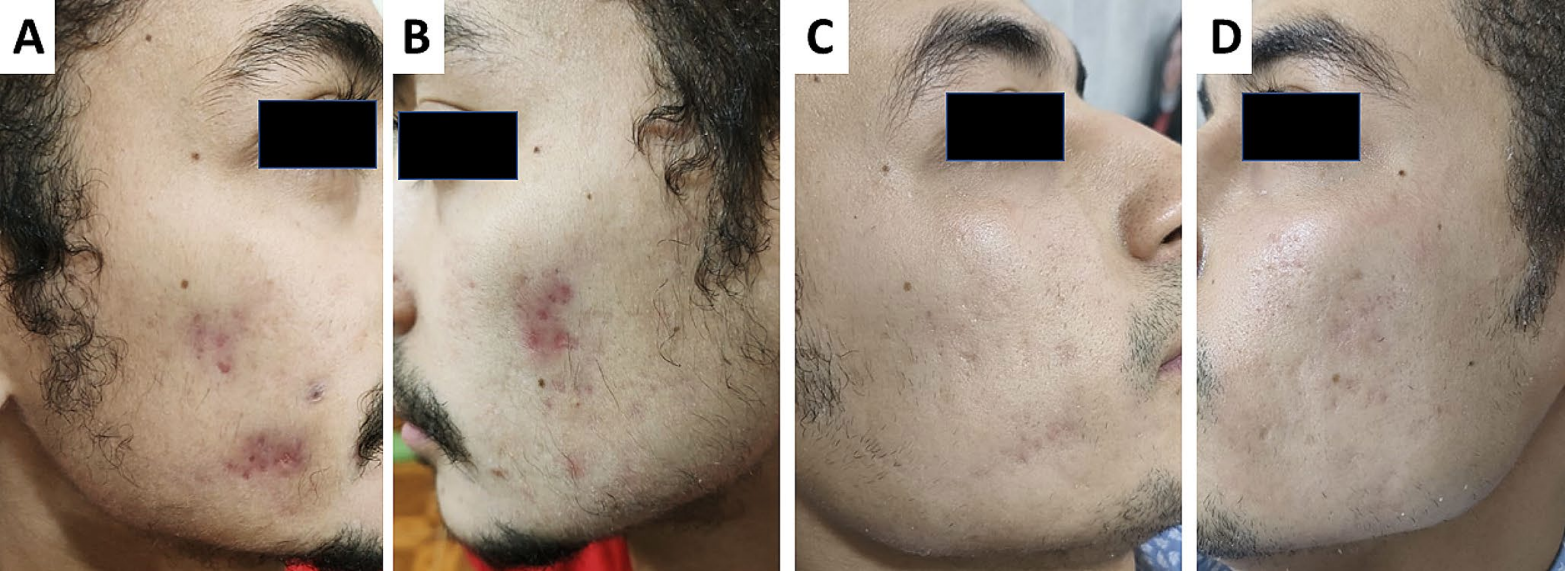
Patient with excellent response following treatment with isotretinoin and long-pulsed ND-Yag: A&B: before treatment, C&D: after treatment

**Fig. 2:**
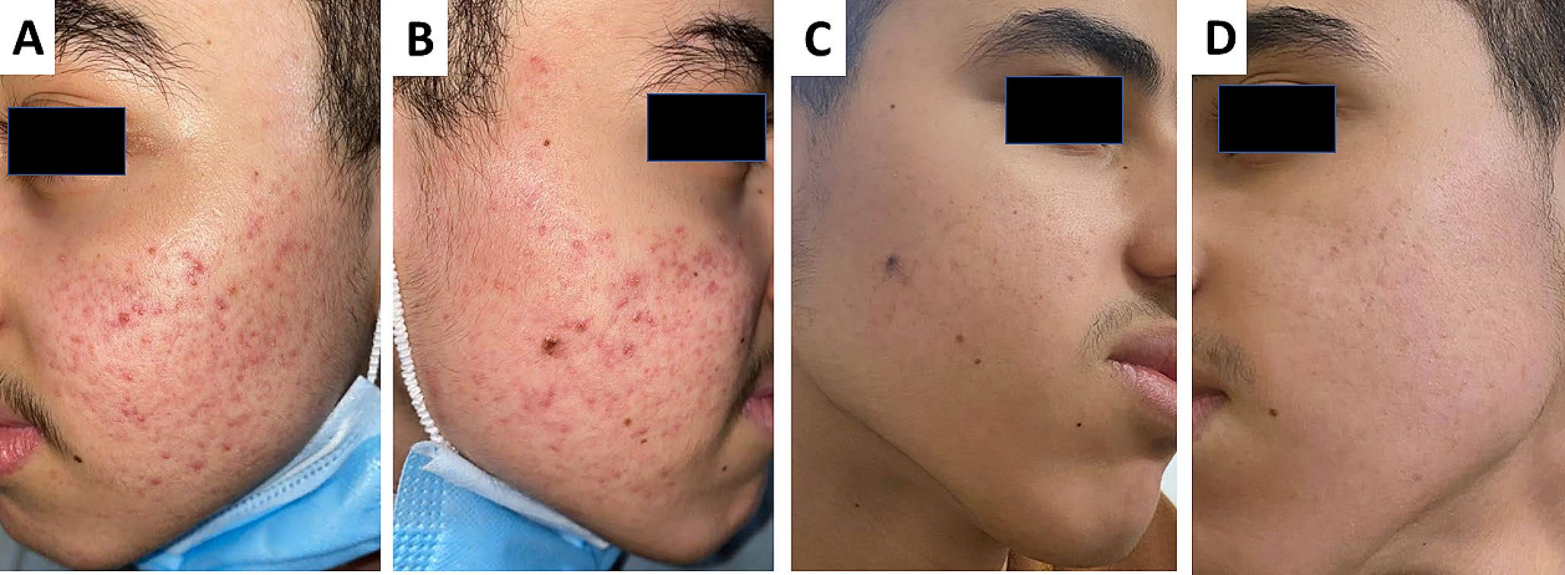
Patient with excellent response following treatment with isotretinoin and long-pulsed ND-Yag: A&B: prior to treatment, C&D: following treatment

Regarding clinical improvement (eight 33% versus two 8%) patients showed excellent improvement(figure 1 & 2), (seven 29% versus three 13%) patients showed good improvement, (five 21% versus nine 38%) patients had moderate improvement, and (four 17% versus ten 42%) patients with mild responses when assessed by the investigators following the end of treatment sessions in groups (A) and (B), respectively.

Despite the significant improvement in both groups, there were significantly superior results in group (A) regarding the clinical improvement, the CEAS, and the optical density of the erythema in comparison to group (B). Table (2).

The laser treatment sessions only led to mild discomfort during the procedure and temporary facial redness that lasted for a few hours.

**Table 2:** Demographic data and clinical response of the studied groups

	Group A	Group B	
Age (Mean ± SD)	25.13 ± 5.21	20.67 ± 4.53	0.004 ^a^
Gender Male Female	9 (37.5%) 15 (62.5%)	13 (54.2%) 11 (45.8%)	0.247 ^b^
Degree of improvement Excellent Good Moderate Mild	8 (33.3%) 7 (29.17%) 5 (20.83%) 4 (16.6%)	2 (8.3%) 3 (12.5%) 9 (37.5%) 10 (41.7%)	0.03* ^**b**^
CEAS before (Mean ± SD)	3 ± 0.76	3 ± 0.58	0.99 ^a^
CEAS after (Mean ± SD)	1.29 ± 0.98	1.96 ± 0.93	0.03* ^**a**^
	< 0.0001* ^**a**^	0.003* ^**a**^	
OD before (Mean ± SD)	0.485 ± 0.072	0.4978 ± 0.12	0.91 ^a^
OD after (Mean ± SD)	0.278 ± 0.112	0.365 ± 0.021	0.04* ^**a**^
	< 0.0001* ^**a**^	0.005* ^**a**^	

Significance between groups was done using (a) the Mann-Whitney U test, (b) the chi-square test

*Statistically significant difference (*P* ≤ 0.05)

CEAS: Clinical erythema assessment scale OD: Optical Density

## Discussion

The management of post-acne erythema is still a therapeutic obstacle due to the absence of a commonly agreed-upon treatment approach [9].

Regarding isotretinoin, in this study, we found that low-dose isotretinoin alone can significantly decrease PAE. This result can be attributed to the anti-inflammatory effect of isotretinoin by reducing antimicrobial peptides and the toll-like receptor (TLR)-2-mediated innate immune response [22]. Also, low-dose isotretinoin was found to have antiangiogenic effects in patients with erythematotelangeictatic rosacea [23].

In present-day times, lasers have gathered significant attention and recognition within the domains of dermatology and cosmetic practices owing to their prompt efficacy and favorable results [24]. Consequently, various laser methodologies have been employed to specifically target dilated blood vessels in order to enhance the aesthetic outcome of PAE [10].

The effectiveness of Nd: YAG laser in treating acne and PAE is attributed to its anti-inflammatory properties, which are characterized by elevated levels of TGF-β and reduced levels of IL-8 and toll-like receptor (TLR)-2. Additionally, it destroys dilated microcapillaries and sebaceous glands as a result of photothermal effects. Also, it’s an effective treatment strategy for darker skin photo-types [9, 25].

To our knowledge, this is the first investigation to be carried out to assess the 1064 Nd: YAG laser efficacy when combined with low-dose oral isotretinoin (10 mg/day) in the improvement of PAE.

Ibrahim et al.; 2021 [26] examined the efficacy of low-dose isotretinoin (0.25 mg/kg/day) combined with Pulsed Dye Laser PDL (five sessions) in comparison to standard-dose isotretinoin (0.5 mg/kg/day) for the treatment of inflammatory acne, while this study examined a combination of six sessions of long-pulsed 1064 Nd: YAG laser with a low dose of oral isotretinoin (10 mg/day) versus monotherapy with the same dose of isotretinoin in the treatment of PAE.

They reported that the first group revealed a significant improvement in erythema grading compared to the second after six months of treatment. They proposed that the management of pathogenic erythema through the application of two treatment modalities could result in a more favorable resolution of post-acne sequelae, and this approach may lead to a more accelerated and noticeable reduction in inflammation. This agreed with our concept, as we noticed a significant decrease in the mean CEAS at the end of the treatment duration in group (A) compared to group (B).

Despite the 1064-nm Nd: YAG efficacy in treating acne erythema being comparable to that of 595-nm PDL, as reported by Chalermsuwiwattanakan et al.; 2021, their patients exhibited a preference for Nd: YAG over PDL as a result of its relatively lower incidence of adverse effects and reduced pain levels during laser treatment. They also assumed that the different results may be attributed to factors such as the characteristics of participants, laser parameters, the number of treatments, the time between sessions, and the specific brand of laser device utilized [25].

In the current investigation, the observed adverse events consisted of mild discomfort experienced during the treatment sessions as well as transient facial erythema that subsided within a few hours. One of the limitations inherent in our study was the relatively small sample size of participants. Additionally, the limited duration of the follow-up period restricts the ability to gather comprehensive data regarding the impact of the treatment over an extended period.

## Conclusions

The integration of 1064-nm Nd: YAG and low-dose isotretinoin is a secure and effective treatment for acne erythema and should be taken into consideration as an adjuvant or alternative treatment option in persistent PAE patients because it is less painful, more comfortable for the patient, and requires nearly no downtime. Participants with darker skin types experience fewer complications, making it a more favorable condition.

## Data Availability

No datasets were generated or analysed during the current study.
